# Concurrent Primary Hyperparathyroidism and Sarcoidosis in a Patient With Severe Hypercalcemia

**DOI:** 10.7759/cureus.44669

**Published:** 2023-09-04

**Authors:** Mohammed Ayyad, Mansour Khaleel, Maram Albandak, Hadeel K. M. Abedalhameed, Mohab W. J. Najjar

**Affiliations:** 1 Internal Medicine, Al-Quds University, Jerusalem, PSE; 2 Internal Medicine, Al-Makassed Charitable Society Hospital, Jerusalem, PSE; 3 Internal Medicine, Saif Medical Center, Tulkarm, PSE

**Keywords:** endocrinology, parathyroid gland adenoma, adult primary hyperparathyroidism, hypercalcemia, sarcoidosis

## Abstract

Hypercalcemia is a common biochemical abnormality caused by various etiologies, with primary hyperparathyroidism (PHPT) and malignancies being the most common culprits. Differentiating between PTH-dependent and PTH-independent hypercalcemia is crucial in clinical practice. However, in certain clinical contexts, it is important to consider the rare occurrence of two separate conditions causing hypercalcemia simultaneously. Herein, we have described the case of a patient who presented with high serum calcium, a normal PTH level, and histopathological evidence of active granulomatous disease, indicating the presence of both PHPT and sarcoidosis. The coexistence of these conditions poses diagnostic challenges due to their biochemical and clinical similarities. This case highlights the importance of individualized management for patients with concurrent conditions contributing to hypercalcemia. It also emphasizes the need for further research to unravel the underlying interactions between PHPT and sarcoidosis in the context of calcium metabolism. A better understanding of these interactions can guide optimal diagnostic and therapeutic strategies for patients with complex presentations of hypercalcemia.

## Introduction

Sarcoidosis and primary hyperparathyroidism (PHPT) are well-recognized culprits behind hypercalcemia. However, malignancy-associated hypercalcemia and PHPT together constitute about 90% of instances of all causes of hypercalcemia [1]. Sarcoidosis, although occurring less frequently, can contribute to hypercalcemia, making up approximately 5-10% of instances. The underlying mechanism for hypercalcemia in sarcoidosis involves activated macrophages releasing 1-alpha hydroxylase, leading to heightened levels of active vitamin D. This, in turn, triggers the release of calcium from the bones [2-4]. Conversely, hypercalcemia in PHPT is linked to an overabundance of parathyroid hormone (PTH) secretion, which prompts the activation of vitamin D and the reabsorption of calcium from both the kidneys and the small intestines [5].

Hypercalcemia usually doesn't exhibit noticeable symptoms and is frequently asymptomatic. However, in cases of a sudden and severe increase in calcium levels (>13 mg/dL), it can lead to severe symptoms [6]. The primary approach to treating hypercalcemia involves intravenous hydration. Additional therapeutic options that could be taken into consideration comprise bisphosphonates, loop diuretics, surgical intervention, and the use of glucocorticoids for cases associated with granulomatous disorders [7].

We herein report a case of a female patient diagnosed with hypercalcemia who was found to have concurrent sarcoidosis and PHPT. The patient was treated with parathyroidectomy and glucocorticoids with marked improvement. Interestingly, the occurrence of PHPT and sarcoidosis simultaneously in a single patient has been seldom reported in the literature [8]. We aim to provide insight into the clinical and radiological presentation of these patients, as well as the therapeutic approach for both conditions.

## Case presentation

A 59-year-old woman presented to the hospital complaining of generalized weakness for the past two weeks, which was associated with diffuse achy abdominal pain, constipation, and unintentional weight loss for the past few months. The patient also reported experiencing night sweats, diffuse bony pain, arthralgia, and morning stiffness for the past seven months. Her past medical history was significant for hypertension, hyperlipidemia, and type 2 diabetes mellitus.

On examination, the patient was in obvious distress due to pain. She was conscious, alert, and oriented to time, place, and person. Her vital signs revealed a blood pressure of 100/60 mmHg, a heart rate of 95 beats per minute, and an oxygen saturation of 95% on room air. General examination revealed dry mucous membranes, increased skin turgor, and diffuse bone tenderness. The abdominal exam was positive for epigastric abdominal tenderness. The patient had no visible skin rash, oral or genital ulcers, photosensitivity, melena, hematemesis, dysphagia, or dysuria.

Initial laboratory investigations were remarkable for hypercalcemia with elevated creatinine and inflammatory markers as shown in Table 1. Other laboratory values including thyroid function tests were within normal limits. Further workup for malignancy including serum protein electrophoresis (SPEP) and urine protein electrophoresis (UPEP) was within the normal range, which excluded multiple myeloma as a potential cause.

**Table 1:** Summary of the patient's laboratory investigations 25-OH vitamin D: 25-hydroxy vitamin D; ESR: erythrocyte sedimentation rate; ALP: alkaline phosphatase; IgG: immunoglobulin G

Parameters	Result	Normal value
Calcium	13 ↑	8.6-10.2 mg/dL
Phosphorus	2.93	2.5-4.5 mg/dL
Parathyroid hormone	52.9	9-80 pg/mL
25-OH vitamin D	17.6	20-40 ng/mL
ESR	115 ↑	0-15 mm/hr
Creatinine	1.17 ↑	0.6-1.1 mg/dL
ALP	147 ↑	40-130 u/l
Globulin	4.3 ↑	2.5-3.5 g/dL
IgG	2133 ↑	800-1800 mg/dL
Cancer antigen 15-3	53 ↑	6-23.5 u/mL

**Figure 1:**
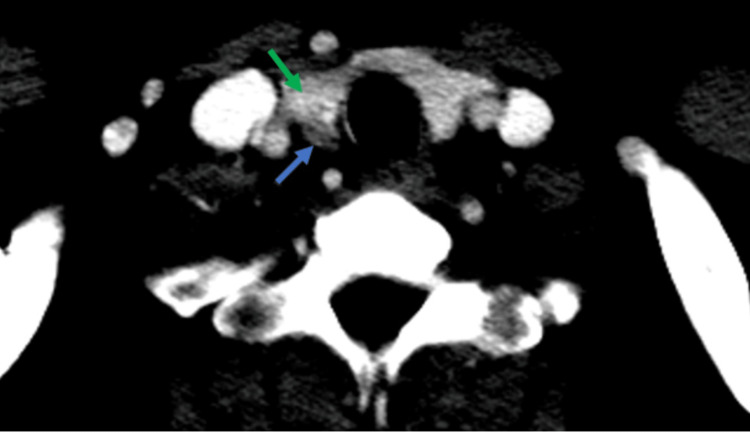
Axial CT scan of the neck with contrast Axial neck CT scan with intravenous contrast depicting a distinct 9 mm oval-shaped nodule (blue arrow) contiguous with the lower aspect of the right thyroid lobe. The nodule exhibits enhancement, albeit to a lesser extent than the thyroid lobe (green arrow). The observed location and enhancement pattern strongly suggest the presence of a parathyroid adenoma. CT: computed tomography

**Figure 2:**
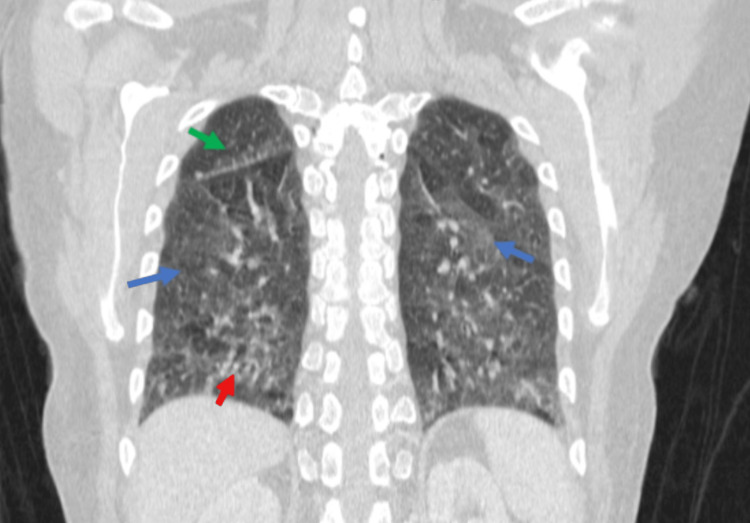
Coronal chest CT scan showing features consistent with sarcoidosis Coronal chest CT scan illustrating characteristic features of sarcoidosis. Ground glass opacities are evident in both lungs (blue arrows), accompanied by micronodules along the peribronchovascular bundles (red arrow), and subpleural distribution of these micronodules along the fissure (green arrow). CT: computed tomography

**Figure 3:**
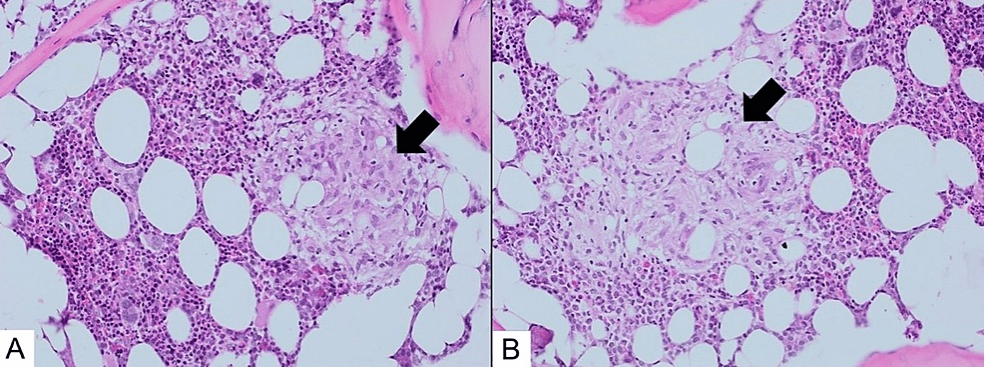
Photomicrograph of bone marrow biopsy showing features consistent with sarcoidosis Bone marrow histopathology (H&E, 20X) depicting numerous non-necrotizing epithelioid granulomas indicative of sarcoidosis (black arrows). H&E: hematoxylin and eosin

Further imaging revealed a 1.2x0.7 cm hypoechoic lesion inferior to the right thyroid lobe on neck ultrasound, which was suggestive of parathyroid adenoma. Subsequently, a sestamibi scan revealed a small focal uptake inferior to the right lobe of the thyroid in the delayed two-hour phase. A whole-body computed tomography (CT) scan confirmed the presence of a small hypodense lesion located inferior to the right lobe of the thyroid (Figure 1). It also detected bilateral ground-glass opacities in the posterior basilar segments of the lungs along with a 7 mm subpleural nodule in the posterior basal side of the left lower lung (Figure 2). Additionally, bone marrow biopsy detected multiple non-caseating granulomas suggestive of sarcoidosis (Figure 3).

**Figure 4:**
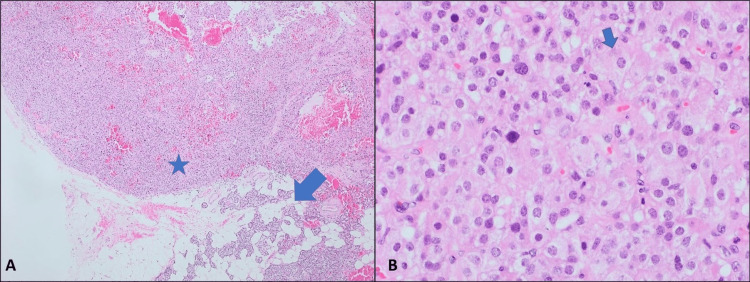
Histopathological specimen of the parathyroid adenoma (A) Histopathological specimen illustrating a distinct proliferation/nodule with neighboring normal parathyroid tissue (arrow), characteristic of adenoma (H&E, 4X). (B) Adenoma cells exhibiting moderate to abundant eosinophilic slightly granular cytoplasm, indicative of oxyphilic variants (H&E, 40X). Chromogranin immunostaining confirmed positivity in adenoma cells (not depicted). H&E: hematoxylin and eosin

Subsequently, the patient was treated with aggressive fluid therapy, bisphosphonates, and calcitonin. Despite these efforts, her symptoms only marginally improved. Consequently, the surgical team opted for a selective parathyroidectomy of the right inferior gland. The tissue resected was sent for histopathological evaluation and showed adenoma cells of the oxyphilic variant with positive staining for chromogranin and negative staining for thyroid transcription factor-1 (TTF-1), confirming the diagnosis of parathyroid adenoma (Figure 4).

Postoperatively, the patient’s symptoms resolved with subsequent normalization of her calcium levels at 8.12 mg/dL and a PTH level of 9.6 pg/mL. The patient was treated with calcitriol and calcium. Additionally, she was started on corticosteroids and hydroxychloroquine to manage her sarcoidosis-induced arthropathy. On follow-up, the patient reported a marked improvement in her condition and resolution of her symptoms.

## Discussion

Hypercalcemia is a commonly encountered biochemical anomaly with various etiologies. Nevertheless, over 90% of all instances of hypercalcemia can be attributed to either PHPT or malignancies [1]. The signs and symptoms of hypercalcemia lack specificity, contingent upon variations in serum calcium concentration, the rate of its elevation, and the underlying etiology. Generally, mild hypercalcemia within the 11-11.5 mg/dL range tends to remain asymptomatic, with no notable symptoms. On the contrary, a sudden and severe rise in calcium levels (>13 mg/dL) can trigger symptoms such as lethargy, stupor, and in extreme scenarios, coma and death [6].

In clinical practice, distinguishing the cause of hypercalcemia involves assessing corrected serum calcium and PTH levels. A heightened corrected serum calcium level, coupled with an inappropriately elevated PTH concentration, indicates a PTH-dependent form of hypercalcemia, which is often associated with PHPT. Conversely, when hypercalcemia occurs alongside suppressed or low-normal PTH values, it suggests a PTH-independent form of hypercalcemia, which may be attributed to conditions such as granulomatous disorders and malignancy among other causes [9-11].

Encountering the concomitant presence of two distinct conditions giving rise to hypercalcemia is a seldom-documented occurrence in the existing literature. In situations where an unusually elevated serum calcium level coincides with normal or high-normal PTH levels, recognizing the potential for dual pathology becomes essential for achieving a precise diagnosis and implementing appropriate interventions [6]. In PHPT, the excessive release of PTH results in hypercalcemia by enhancing both renal calcium reabsorption and bone resorption [12]. Moreover, it stimulates the synthesis of 1,25-dihydroxyvitamin D3 (calcitriol) in the kidneys, thereby increasing calcium absorption in the gastrointestinal tract [13]. On the contrary, hypercalcemia in sarcoidosis, which is seen in 5-10% of patients, mainly arises from the excessive intrinsic generation of calcitriol by alveolar macrophages located in the tissue of the affected lungs. Furthermore, sarcoid granulomas can also generate parathyroid hormone-related protein (PTHrP), further contributing to elevated calcium levels [2-4].

The presence of a drastically elevated serum calcium in our patient, accompanied by a normal PTH level, raised the possibility of a coexisting PHPT and another PTH-independent cause of hypercalcemia. While isolated PHPT is associated with high PTH, the concurrent presence of a PTH-independent etiology could explain the unusually normal PTH concentration observed in our patient. Furthermore, the presence of a low vitamin D despite normal PTH levels further raises the suspicion of a dual pathology of hypercalcemia, as low vitamin D levels are often associated with more severe PHPT, characterized by higher PTH levels [14].

Following a thorough assessment, several potential causes of hypercalcemia were carefully considered and subsequently ruled out, taking into account the patient's medical background, medication history, and laboratory tests. Conditions including hyperthyroidism, tertiary hyperparathyroidism, familial hypocalciuric hypercalcemia, drug-induced hypercalcemia, and other uncommon disorders such as milk-alkali syndrome were meticulously considered and ruled out. However, based on the patient's history, additional imaging studies, and histopathological examination, the two most probable culprits for the hypercalcemia were identified as PHPT and sarcoidosis.

Nevertheless, the precise pathophysiology of calcium metabolism when both sarcoidosis and PHPT coexist remains unclear, mainly due to the scarcity of reported cases in the medical literature where these two conditions occur simultaneously [12,13,15,16]. Additional research and thorough investigation are imperative to unveil the underlying mechanisms and interplay between these conditions concerning calcium metabolism.

The biochemical resemblances between these two conditions can pose challenges in discerning their respective effects on calcium metabolism. Moreover, it is important to acknowledge that sarcoid granulomas have displayed resemblances to parathyroid adenomas in imaging modalities like ultrasound and thallium-technetium scans. This resemblance further adds complexity to the diagnostic process [17].

Surgical excision of the hyperfunctioning parathyroid tissue constitutes a definitive treatment approach for PHPT. While debates may arise regarding the most suitable timing for surgical intervention, there is widespread recognition of the effectiveness of surgery in addressing this condition [11]. Nonetheless, corticosteroids are frequently employed to manage hypercalcemia in sarcoidosis. Additionally, hypercalcemia serves as a criterion to initiate steroid therapy, even in situations where only mild symptoms are evident [18]. In instances where both conditions coexist, managing hypercalcemia can be intricate, and the response to treatment may vary [15].

Our patient underwent a parathyroidectomy, resulting in the amelioration of her symptoms and the reinstatement of physiological calcium and PTH levels. Subsequent long-term follow-up has not shown any recurrence of symptoms or the underlying diseases. Based on these observations, we conclude that the hypercalcemia in our patient was mainly due to PHPT, with minimal or negligible contribution from her sarcoidosis. This case underscores the significance of tailored management and the need to take into account the unique characteristics of each patient when addressing complex scenarios involving the coexistence of multiple conditions.

## Conclusions

In conclusion, we've presented a unique case characterized by hypercalcemia attributed to the concurrent occurrence of PHPT and sarcoidosis, with significant clinical implications. When addressing patients displaying PHPT and severe hypercalcemia, the differential diagnosis becomes intricate. It's essential to entertain the possibility of multiple diseases contributing to hypercalcemia, particularly when extremely elevated calcium levels coincide with normal PTH values. Additionally, when conventional treatments targeting a specific etiology fail to alleviate hypercalcemia, it is imperative to explore the potential contribution of other underlying conditions. This underscores the necessity for a thorough evaluation and diagnostic strategy in such scenarios, ensuring precise identification of the underlying factors contributing to the hypercalcemic state.
